# Neonatal Thoracic Infection with *Mixta*

**DOI:** 10.1016/j.infpip.2021.100114

**Published:** 2021-01-13

**Authors:** Christo Tsilifis, Lucia Pareja-Cebrian

**Affiliations:** a Paediatric Immunology and Infectious Diseases, Great North Children's Hospital, Royal Victoria Infirmary, Newcastle Upon Tyne, United Kingdom; b Translational and Clinical Research Institute, Faculty of Medical Sciences, Newcastle University, Newcastle Upon Tyne, United Kingdom; c Department of Microbiology and Virology, Royal Victoria Infirmary, Newcastle Upon Tyne, United Kingdom

**Keywords:** Mixta, Erwiniaceae, Neonate, Nosocomial

## Abstract

The *Erwiniaceae* are a family of gram-negative, aerobic coliforms which are pathogenic to a number of plants. Recently described within this family are the *Pantoea*, strains of which have been associated with infection in immunocompromised children and post-surgical meningitis but also colonisation of a healthy human subject's gastrointestinal tract, as well as a variety of agricultural diseases. In 2015, a further clade of this family was established as the genus *Mixta*.

In this case report, we describe infection of the pleural space and lung parenchyma with members of *Mixta* in a term neonate following an anastomic leak post-primary repair of congenital trache-oesophageal fistula, causing a respiratory and cardiovascular deterioration. *Mixta* were identified by MALDI-TOF. The child made a full recovery with use of intravenous piperacillin-tazobactam.

The *Mixta* genus must be added to a list of opportunistic pathogens responsible for infection following perforation of the gastrointestinal tract.

## Introduction

The *Erwiniaceae* are a family of gram-negative, aerobic coliforms which are pathogenic to a number of plants [1]. Recently described within this family are the *Pantoea*, strains of which have been associated with infection in immunocompromised children [2] and nosocomial infection such as central venous line-associated bacteraemia and post-surgical meningitis [3], but also colonisation of a healthy human subject's gastrointestinal tract, as well as a variety of agricultural diseases [4]. In 2015, a further clade of this family was established as the genus *Mixta*.

## Case report

A term neonate was admitted to the general paediatric intensive care unit (PICU) shortly following birth for pre-operative care, having been postnatally diagnosed with tracheoesophageal fistula after a dusky episode. He was initiated on intravenous benzylpenicillin and gentamicin for suspected neonatal sepsis.

He underwent primary repair on day 1 of life and was nursed on PICU post-operatively. Pre-operative echocardiography showed a structurally and functionally normal heart. His repair was complicated by a high upper pouch giving rise to a tight anastomosis. He received a single dose of Co-amoxiclav as peri-operative surgical prophylaxis.

He returned to PICU intubated with a right-sided anastomotic drain in situ. He was commenced on total parenteral nutrition via percutaneous longline on day two of life. Intravenous antibiotics were stopped on day three of life, and continuous feeds of breastmilk were commenced via a trans-anastomotic tube.

The patient was then extubated on day seven of life. Shortly after extubation, he developed respiratory and cardiovascular instability with rising FiO_2_, reduced air entry on the right hemithorax and hypotension. Chest radiography revealed a right tension pneumothorax which was treated emergently with intubation and aspiration of approximately 25 mls of yellow fluid and air. A second chest drain was subsequently inserted, and the patient was commenced on intravenous piperacillin/tazobactam and fluconazole due to suspicion of anastomotic leak. Biochemical analysis of the pleural aspirate showed presence of white blood cells, a raised lactate dehydrogenase (3495 units/litre) and high protein (15g/decilitre) consistent with an exudative process.

He continued to develop features of sepsis with hypotension, tachycardia and disseminated intravascular coagulopathy, and so teicoplanin was added to cover for central-line associated Gram-positive organisms. Laboratory investigations demonstrated a neutrophilic leukocytosis, with peak white blood cell count 38.4 x10^9^ cells/litre, as well as a raised C-reactive protein (peaking at 179 mg/litre). His serum lactate peaked at 4.0mg/litre. He did not need vasoactive infusions.

Endotracheal secretions, pleural aspirate and nasopharyngeal aspirate taken on the day of his deterioration yielded *Mixta spp* on culture; antibiotic sensitivities are summarised in Table I. Blood cultures taken at the time of deterioration yielded no growth after five days. There were no faecal cultures sent. Antibiotics continued from day seven of life until day 23 of life, with a further two days of fluconazole given after this. On day 13 of life, endotracheal secretions were collected to investigate control of infection and eradication of the pathogen. These isolated *Pseudomonas aeruginosa*, presumptively colonising the endotracheal tube.

**Table I:** Summary of microbiological cultures.

Day of life	Culture	Growth	Sensitivities	Resistances
7	Endotracheal secretions	*Mixta spp.*	Amikacin, ciprofloxacin, co-amoxiclav, co-trimoxazole, piperacillin/tazobactam	Amoxicillin
7	Pleural fluid	*Mixta spp.*	As above	As above
7	Nasopharyngeal aspirate	*Mixta spp.*	As above	As above
7	Peripheral blood	No growth	-	-
13	Endotracheal secretions	*Pseudomonas aeruginosa*	-	-

The patient was subsequently extubated on day 21 of life and continued to feed via trans-anastomotic tube. He was discharged from the PICU on day 27 of life.

## Discussion

The genus *Mixta* is a newly described clade of the *Erwiniaceae*, a family of Gram-negative bacilli, taxonomically separated from *Pantoea* in 2018 [1] by findings including inability to produce yellow pigment. Due to the recent definition of *Mixta*, there is limited data on the role in human pathology of these organisms specifically.

*Pantoea* have previously been described as pathogenic in humans, often in the context of nosocomial infection though reports exist of soft tissue and articular infection following penetrating trauma by vegetation [2,5]. *Pantoea agglomerans* is the most commonly isolated species in humans; Cruz *et al.* [2] published a series of 53 paediatric cases of *P. agglomerans* isolated from usually sterile sites; notably, the largest proportion of positive sterile-site cultures (21/53) were central venous line-associated bacteraemias, of which 14/21 were polymicrobial. The authors identified 26 sputa with growth of *P. agglomerans*, although only one grew this repeatedly; the others were polymicrobial. There is no previously described pleural infection with *Pantoea*.

Despite the temporal relationship between isolation of this pathogen and the patient's anastomotic leak, it is difficult to identify a definitive source or reservoir. Previous endotracheal and nasopharyngeal specimens had not isolated this organism.

Contamination of breastmilk and subsequent infection via the dehisced anastomosis remains a possibility. There are no reports of breastmilk contaminated with *Pantoea* or *Mixta*, though interestingly *P. calida* has previously been isolated from powdered milk formula [6]. In our patient, breastmilk was not cultured. *P. intestinalis* has been isolated from human faeces previously [4], though this report does not include clinical details or the subject's age. The lack of concurrent faecal culture in our patient makes drawing conclusions challenging.

It remains possible that the organism identified represented colonisation, and the clinical phenotype resulted from systemic inflammatory response rather than true infection; however, the improvement with antibiotics makes this less likely.

## Conclusion

We describe the first case of thoracic infection with *Mixta*, in a neonate with contamination of milk into the mediastinal space. The patient had a clinical deterioration compatible with sepsis and responded to fluid resuscitation and antibiotic therapy with piperacillin/tazobactam and antifungal cover with fluconazole. The provenance of this organism is unclear, though contamination of breastmilk is a possibility. Identification of risk factors for other patients is challenging, though experience of closely-related pathogens such as *Pantoea* would suggest that invasive procedures and immunocompromise may contribute to risk of infection.

## Funding sources

CT is funded by the Job Research Foundation.

## Conflicts of interest

No conflicts of interest have been disclosed by the authors.

## Authorship

LP conceived the project and provided clinical data and critical review. CT gained informed consent, collated the relevant data and produced the manuscript. Both authors contributed to writing the final version of the manuscript and approved the final version.

## Consent

Written informed consent has been given by the patient's parent for publication of this case report and accompanying data and has been retained by the authors. A copy of the written consent is available for review by the Editor-in-Chief of this journal on request. Ethical committee review was not sought due to the manuscript detailing retrospective clinical and laboratory data following the patient's recovery.
